# Higher frequency of Congenital Hypothyroidism among Newborns, District Dera Ghazi Khan-Punjab, Pakistan: A case control study

**DOI:** 10.12669/pjms.37.5.4086

**Published:** 2021

**Authors:** Abdul Rehman Khokhar, Abdul Majeed Cheema

**Affiliations:** 1 Dr. Abdul Rehman Khokhar Professor of Physiology, Department of Physiology, D.G. Khan Medical College, Dera Ghazi Khan, Punjab, Pakistan; 2 Dr. Abdul Majeed Cheema Professor of Physiology IMBB, University of Lahore, Pro VC Leeds University, Lahore, Pakistan

**Keywords:** Congenital hypothyroidism, Neonatal TSH, Neonatal total T4

## Abstract

**Objectives:**

The study objective was to establish serum TSH cut off value for diagnosis of new case of congenital hypothyroidism and to estimate frequency of Congenital Neonatal Hypothyroidism.

**Methods:**

A case control study was conducted at DHQ Teaching Hospital of DG Khan Medical College, Dera Ghazi Khan during 2020 to establish reference values of TSH and T4 for study population. Sample size was calculated by classical sample size calculation formula Cochran WG 1977 sampling technique. A group of 30 neonates of normal, healthy, euthyroid mothers was taken as Neonatal Control Group to estimate levels of TSH and total T4 in normal neonates. Neonatal Study Group was neonates of hypothyroid mothers (n=75). Simple random sampling technique was applied.

**Results:**

Mean (mean ± SD) Serum TSH levels of Neonatal Control Group were found to be 3.58 ± 03.09 mIU/l. Mean Serum TSH levels among Neonatal Study Group were found to be 6.88 ±12.95 mIU/l and serum total T4 were found to be 16.78 ± 6.96ug/dl on 3-7 days of life. Serum total T4 (mean ± SD) levels of Neonatal Control Group were 9.73 ± 03.39 ug/dl. Neonatal serum TSH more than 15mIU/l was taken as cut off value to diagnose a case of CNH. So, frequency of CNH was 8% among neonates of study group.

**Conclusions:**

The TSH cut off value of >15mIU/l was established for case detection of CNH. Our findings of CNH in district Dera Ghazi Khan (8%) are the highest frequency of CNH reported so far in Pakistan.

## INTRODUCTION

Congenital neonatal hypothyroidism (CNH) is a common cause of mental retardation in children. This is fortunately curable. Fetuses of mothers, having hypo-thyroxinemia, may present with fetal goiter and fetal hypothyroidism. Enlarged thyroid gland (goiter) in fetus just like in adults may be associated with euthyroidism, hypothyroidism or hyperthyroidism.^1^

The incidence of CNH was estimated 1:2500 to 1: 4000 live births worldwide. The major maternal causes of CNH are maternal iodine deficiency, maternal intake of anti-thyroid drugs, excessive iodine intake. Fetal causes may be fetal thyroid dysgenesis, fetal hormonogenesis defect (Pandered syndrome), autoimmune thyroid disease in the fetus, fetal TSH receptor mutation or fetal thyroid tumors.^2^

Anti-thyroid drug administration to treat maternal hyperthyroidism may induce fetal hypothyroidism with fetal goiter. Thyroid dysfunctions are reported in fetus of pregnant women with Grave’s disease, taking anti-thyroid medication, that range from fetal hypothyroidism, fetal goiter, and neonatal thyrotoxicosis.^3^ It is extremely difficult to make prenatal diagnosis of fetal thyroid status. The best way is to avoid maternal hypothyroidism. It is recommended to keep maternal circulating TH levels in upper limit of normal range.^4^

During post-natal period high TSH concentration results in increased serum T4 and, increased T4 conversion into active T3 and reduction in rT3 levels. Adequate iodine supply is essential for synthesis of thyroid hormones during prenatal and postnatal period.^5^

Thyroid hormones are essential for maturation of many tissues including brain, heart, lungs, skeleton and intestine. They are also required for non-shivering thermogenesis. Tissue maturation is dependent on thyroid hormones and maturation of local deiodinase system. Post-natal period has dynamic changes in thyroid hormones secretion and metabolism. These are needed for neonatal adaptation to changing demand of post-natal life.^6^

Primary congenital neonatal hypothyroidism may be caused by goiter, ectopic thyroid or athyrosis. Family history of adulthood onset thyroid disease in mother has no correlation with risk of congenital neonatal hypothyroidism. During newborn screening for CNH, 2% cases have history of maternal autoimmune thyroid disease. TSH receptor auto-antibodies transfer to fetus are contributing factors.^7^

Secondary CNH is a central defect of Hypothalamus-Pituitary origin. It’s is associated with deficiency of pituitary hormones. CNH has checklists of twelve clinical features i.e., prolonged jaundice, sleepy, inactivity, delayed passage of meconium, constipation, poor feeding, umbilical hernia, goiter, microcephaly, large Fontanilla, low birth weight, poor weight gain. These symptoms are non-specific and diagnosis may be missed.^8^

Each screening program should develop its own TSH cut off values to detect CNH. If TSH>30mu/l or 15 mu/l of whole blood, infant serum testing should be carried out to confirm the diagnosis. In case of borderline results i.e. low T4 but TSH below cutoff values, repeat screening specimen is recommended after two weeks. Premature neonates showed hypo-thyroxinemia, delayed TSH elevation which are difficult to distinguish, so FT4 is preferred in such cases.^9^

The increasing incidence of CNH over last 20 years is still unresolved controversy. Whether the increase is real or high premature birth rate or lower screening test cut off values, racial, ethnic differences of population are not well understood.^10^ T4 therapy should be started promptly after diagnosis of CNH. Neonate should be euthyroid as early as possible. An inverse relationship was reported between age at diagnosis and I.Q of the infant. Later onset of therapy resulted in neurological defect.^11^

The neonatal screening protocol for congenital neonatal hypothyroidism, that serum TSH estimation alone cannot identify all cases of Congenital Hypothyroidism, so expanded neonatal screening along with simultaneous estimation of Serum TSH and T4 levels is recommended. Neonatal Serum T4 estimation in newborn screening sample may prevent delayed diagnosis among subjects having no early and non-specific symptoms.^12^ The study objective was to establish serum TSH cut off value for diagnosis of new case of congenital hypothyroidism and to estimate frequency of Congenital Neonatal Hypothyroidism of study population of a remote district of Pakistan.

## METHODS

This study was conducted by Department of Physiology, IMBB University of Lahore in collaboration with departments of Gynecology & Obstetric and Pediatric Medicine of Teaching DHQ Hospital Dera Ghazi Khan during 2020. This was a cross control, hospital-based study. Simple random sampling technique was applied. The study sample size was calculated by Classical Sample size formula Cochran WG 1977, sampling technique, John Wiley New York.^13^ Study participants were two groups: A group of already diagnosed hypothyroid mothers was identified and recruited to estimate frequency of CNH among neonates born to hypothyroid mothers. Mothers were newly diagnosed cases of hypothyroidism in last trimester of pregnancy. The Neonatal Study Group participants were seventy-five (n=75). Another group of neonates of normal, healthy pregnant mothers was recruited as Neonatal Control Group. Study participants of neonatal control group were thirty (n=30). Ages of neonates of both groups were 5-7 days of life. Pregnant mothers suffering from pre-eclampsia, eclampsia, hypertension, ante-partum hemorrhage, Diabetes, oligo-hydromnios, twin pregnancy, anti-thyroid medication, twin pregnancy and seriously ill were excluded from the study. Premature, seriously ill neonates were also excluded. The written informed consent was obtained from subject’s parents and confidentiality of data was maintained. Blood samples of all subjects were taken on 5-7 days of life aseptically to estimate serum TSH and serum total T4 levels. Blood was allowed to clot for 15-20 minutes. Then it was centrifuged for ten minutes at 5000 rpm. Serum was sucked from centrifuge tube and transferred into serum cups and stored at -20ºC.

Serum TSH levels and total T4 Levels were estimated by Chemi-illumination micro particle assay (C-MIA) ARCHITECT fully automated ELISA, Abbott Diagnostics. The highest serum TSH level value of Neonatal Control Group was practiced to label a case of congenital neonatal hypothyroidism and considered as standard TSH cut off value as a reference value for Pakistani population. Independent variables were neonatal serum TSH and neonatal T4, age, sex and data were analyzed by using SPSS version 18.0.

### Ethical Approval & Consent to participate

The study protocol was approved by Ethical Review committee of DGKMC, Dera Ghazi Khan vide no. 37/2018; dated 15-8-2018 and Institutional Review Board of University of Lahore. Written, informed consent form was dully signed by parents each participant.

## RESULTS

Seventy-five neonates of hypothyroid mothers were enrolled as Neonatal Study Group (n=75) and newborn babies of Maternal Control Group were labeled as Neonatal Control Group (n=30). Among Neonatal Study Group, out of 75, thirteen lost during follow up and only sixty-two were available for data analysis (n=62). Among Neonatal Control Group (n=30) three neonates lost during follow up and twenty-seven were available for data analysis.

Age (mean ± SD) of Neonatal Study Group was 4.67 ± 1.10 days and weight (mean ± SD) of neonates was 2.90 ± 0.48 Kg and, weight ranges between 2.0 – 4.0 Kg. Age (mean ± SD) of Neonatal Control Group was 3.55 ± 0.97 days and weight (mean ± SD) of neonates was 2.94 ± 0.42 Kg and, weight ranges between 2.5 – 4.0 Kg (Table-I).

**Table-I:** Demographic Parameters of Neonatal Study and Neonatal Control Group participants.

Name of Group	Neonatal		Parameters	

	Age (Days)	Sex	Gestational Age	Weight (Kg)
Neonatal Study group n=62	4.67 ± 1.10	Female n=47(75.8%) Male n=15(24.2%)	>37- 40 weeks n=61 (98.4%) >40 Weeks n=01 (1.6%)	2.90±0.48
Neonatal Control group n=27	3.55 ± 0.97	Female n=13(48.15%) Male n=14(51.85%)	>37-40 weeks n=26(96.27%) >40 weeks n=01(3.73%)	2.94±0.42

Neonatal serum TSH levels of both neonatal groups were estimated. Serum TSH (mean ± SD) levels of Neonatal Study Group were 6.88 ± 12.95 mIU/l, ranges between 0.100–100.0 mIU/l. Serum TSH (mean ± SD) levels of Neonatal Control Group were 3.58 ± 03.09 mIU/l, ranges between 0.260 – 14.8 mIU/l (Table-II).When independent sample t-test was applied to compare serum TSH levels of Neonatal Study Group with TSH levels of Neonatal Control Group (TSH vs TSH) , SEM for Neonatal Study Group was 1.645 and SEM for Neonatal Control Group was 0.595, the results were which were non- significant statistically (P>0.05). When Independent t-test was applied for equality of means and variances F-distribution was 1.68 (P>0.05) which was non- significant statistically and t-test for equality of means was also non- significant statistically P > 0.05 (Table-II).

**Table-II:** Thyroid tests profile of both Neonatal Study group and Neonatal Control Group.

Parameter	Group	Number	Mean ±SD	SEM	Range	P-Value
TSH (Neonatal)	Control	n=30	3.58 ± 03.09 mIU/l	0.595	0.260 – 13.02	0.06
	Study	n=75	6.89 ± 12.95mIU/l	1.645	0.100 - 100	
Total T4(Neonatal)	Control	n=30	9.73 ± 3.39 ug/dl	0.652	1,180 – 14.81	0.000
	Study	n=62	16.78 ± 6.96 ug/dl	0.883	2.130 -24.00	

Serum total T4 (mean ± SD) levels of Neonatal Study Group were 16.78 ± 6.96 ug/dl/, ranges between 2.130 –24 .00 ug/dl. Serum total T4 (mean ± SD) levels of Neonatal Control Group were 9.73 ± 03.39ug/dl, ranges between 1.180 – 14.81 ug/dl (Table-II). When independent sample t-test was applied to compare serum total T4 levels of Neonatal Study Group with TSH levels of Neonatal Control Group (tT4 vs tT4), SEM for Neonatal Study Group was 0.883 and SEM for Neonatal Control Group was 0.652, the results shown were highly significant statistically (P < 0.001) (Table-II). When Independent t-test was applied for equality of means and variances F-distribution was 21.94 (P > 0.001) which was highly significant statistically and t-test for equality of means was also highly significant statistically P < 0.001 (Table-II).

When serum TSH levels of Neonatal Study Group (n=62) and Neonatal Control Group were grouped into five classes on the basis of serum TSH levels of neonates. Maximum numbers of neonates from Neonatal Study Group 84.6% (55/62) serum TSH levels were below10 mIU/l. Among Neonatal Control Group 100% (27/27) serum TSH levels were below15 mIU/l. Among Neonatal Study Group 8.06% (05/62) participants’ serum TSH levels fall in range of >16-20 mIU/l. As TSH cut off value for diagnosis was > 15 mIU/l for current study so 8% prevalence of congenital Hypothyroidism shown among participants of Neonatal Study Group, and serum T4 levels less than 6.5 ng/dl was a confirmatory test for diagnosis (Table-III).

**Table-III:** Comparison of TSH values of Neonatal Study Group vs Neonatal Control Group for frequency of congenital neonatal hypothyroidism among neonates.

Levels of Serum TSH	Study Group n=62	Control Group n=27
(mIU/l)	Number of neonates (%)	Number of neonates
1--------------15	57 (87.6%)	27 (100%)
15-------------20	03 (4.84%)	0
20	02 (3.2%)	0

Total cases of congenital hypothyroidism = 8%.

## DISCUSSION

This was a pioneer study on congenital neonatal hypothyroidism in Pakistan to establish TSH reference value. No reference TSH cut off values were available for diagnosis of CNH population of Pakistan. The incidence of congenital hypothyroidism reported among children born at Agha Khan University Hospital Karachi after 20 years of neonatal screening. They reported serum TSH cut off value of more than 13 mIU/l to diagnose a case of congenital hypothyroidism.^14^ Karachi city is a coastal region of Pakistan that is different geographically from our study area which is a sub-mountainous region. Anjum et al reported congenital hypothyroidism after a hospital-based study, among children of Lahore city. Their study showed serum TSH cut off value of more than 20mIU/l to diagnose a case of congenital hypothyroidism.^15^ Ahamd et al. reported incidence of congenital hypothyroidism among neonates of Lahore city and reported serum TSH cut off values were more than 30 mIU/l, to label a case of congenital neonatal hypothyroidism.^16^

Researchers reported three different TSH values for diagnosis of congenital hypothyroidism. No reference TSH up to date cut off value was available.^14^-^16^ Our study was conducted to establish a reference TSH cut off value for Pakistani population from a district of Southern Punjab and for estimation of frequency of congenital neonatal hypothyroidism. Our study also estimated serum total T4 reference values of our neonatal population for diagnosis of case congenital hypothyroidism which was first time in Pakistan. Our neonatal T4 reference values for Neonatal Control Group are less than the international reference values.

Neonatal screening is a part of standard child care internationally. In 1970 a screening program was started to detect congenital hypothyroidism during neonatal period. Later on, it was adopted by most of countries of the world. Serum TSH cut off value 17–19.9 mIU/l, from neonatal screening data and follow up for four-year duration from Ontario-Canada.^17^ Anastasovska et al. reported TSH cut off values were > 15 mIU/l before 2010 and thereafter now TSH cut off values > 10 mIU/l are utilized for diagnosis of a case of congenital hypothyroidism. Their study reported five times higher incidence of congenital hypothyroidism form Vardar region as compared to other regions of Macedonia which is geographically mountainous and sub mountainous areas, similar to our study area.^18^

Out of the total birth population of 127 million worldwide, 25% babies undergo neonatal screening for Congenital Hypothyroidism and 75% are from developing countries where only clinical finding of congenital neonatal hypothyroidism are further evaluated al. Family and health professional need to be engaged in orientation of population in poor and underdeveloped nations of the world. South Asian Region countries (SARC) health care is insufficient.^19^

Park and Yoon reported congenital hypothyroidism in infants from South Korea. Their study was retrospective, ten-year duration (2005-2015) and sample size was fifty-one (n=51) selected after neonatal screening for congenital hypothyroidism. Study subjects were of age 2-4 days (Full term), seven days (Preterm). Serum TSH Cut off value > 20 mIU/l or FT4 levels < 0.9 ng/dl for diagnosis of a case congenital neonatal hypothyroidism. Our study finding of serum TSH cut off value and serum T4 levels were contrary to Park and Yoon.^20^

Adachi et al reported mass screening of newborns for congenital hypothyroidism for central origin by FT4 estimation of blood sample on filter paper from Japan. Mass neonatal screening system of Kanagawa prefecture, a central region of Japan screened 70,000 new born during ten years (1999-2008), blood sample was taken on 4-7 days of life and TSH and FT4 estimation was done. TSH cut off value > 30 mIU/l or FT4 level < 0.7ng/dl (9.0 Pmol/l) subjects were sent for further examination by pediatric endocrinologist and onset of therapy. The incidence reported was 1: 3472 among newborn of Kanagawa prefecture. Our study TSH cut off value and T4 value for diagnosis of CNH were contrary to Adachi et al.^21^

Park et al. reported predictors of congenital hypothyroidism in children from Ajou University Hospital Korea. Mass screening program practice serum TSH cutoff value > 10 mIU/l and Low FT3, Low T3 levels for diagnosis of congenital hypothyroidism. Our study findings of TSH cut off value and T4 levels were contrary to Park et al. due different geographical features and dietary habits.^22^

Nagasaki et al reported guidelines for mass screening of congenital hypothyroidism (2014 revision). Japan is a leading country in iodine consumption in the world and iodine deficiency is very rare in Japan’s population. Mass screening for CNH was started in1979 and guidelines were established by Mass Screening Committee of the Japanese Society for Pediatric Endocrinology in 1998 and revised version was updated in 2014, which are in practice up to date. Filter blood sample is being drawn from footpad on 4-6 days of life for TSH estimation. Serum TSH cut off value of > 15 mIU/l is utilized to label a case of CNH. Our study findings of serum TSH cut off established by our Neonatal Control Group were in accordance with Nagasaki et al.^23^

Neonatal Mass Screening in all health facilities of Japan, serum TSH determination is a basic test for diagnosis of neonatal congenital hypothyroidism in first week of life. In North America serum T4 estimation is done in first sample for mass screening, which is followed by serum TSH levels estimation. Simultaneous estimation of both serum TSH and FT4 is also practiced in some regions of Japan.^24^

Mass Screening with TSH is more common in the world due to improved TSH assay sensitivity. TSH and FT4 estimation during mass screening also identify cases of secondary (Pituitary), tertiary (Hypothalamus) and primary (Thyroid) congenital hypothyroidism. Our study practiced simultaneous estimation of serum TSH and T4 levels which was in accordance with international recommendations.^25^

### Limitations of the study

Our study has small sample size, from one district of Punjab. More studies need to be conducted with larger sample from different geographical regions to establish national cut off values for congenital hypothyroid among neonates. Incidence of congenital hypothyroidism may be estimated in the Pakistan.

## CONCLUSION

Serum TSH cut off value of >15mIU/l was established for the case detection of CNH. The highest frequency of CNH (8%) was reported from a remote district Dera Ghazi Khan, Punjab-Pakistan. Early detection of CNH is the greatest advances of twentieth century in child health and preventive pediatrics.

### Authors’ Contributions:

**ARK** Conception /Data Collection/data Analysis / Drafting of Manuscript.

**AMC** Study design, supervise the whole study, Final Approval of manuscript.

Both authors read, approved the final manuscript and responsible for accuracy and integrity of research work.
